# Cognitive Style: Time to Experiment

**DOI:** 10.3389/fpsyg.2016.01786

**Published:** 2016-11-15

**Authors:** Robert C. A. Bendall, Adam Galpin, Lynne P. Marrow, Simon Cassidy

**Affiliations:** Directorate of Psychology and Public Health, School of Health Sciences, University of SalfordSalford, UK

**Keywords:** cognitive style, cognition, visual attention, eye-tracking, neuroimaging, functional near-infrared spectroscopy

Evidence exists that individuals possess habitual ways of approaching tasks and situations associated with particular patterns in cognitive processes including decision making, problem solving, perception, and attention. Such approaches are conceptualized as *cognitive style*, a concept first formally introduced by Allport almost eight decades ago and defined as an individual's typical or habitual mode of problem solving, thinking, perceiving, and remembering (Allport, 1937). The popularity of the concept has since continued to grow, leading to a profusion of applied research and commercial applications in such areas as business, management, and education. Such levels of activity have led to the emergence of more than 70 identifiable models and measures of cognitive (and learning) style (Coffield et al., 2004) and a plethora of related terminology, constructs, and measures of style. Consequently, the field of style has become wildly confusing to both researchers and practitioners, and, perhaps justifiably, has received weighty criticism, most notably from Coffield et al. (2004). Following a broad and detailed systematic review of the most popular models and construct measures, Coffield et al. (2004), together with others (e.g., Curry, 1987; Cassidy, 2004), issued a damning critique of style, noting the failure of the field to offer a consensus on definitions and terminology, construct models and underlying theory, and valid and reliable construct measurement. Such concerns present a major obstacle for continued research and practice in the field.

Cognitive style focuses on the tradition of identification of styles based on individual differences in cognitive and perceptual functioning (Grigorenko and Sternberg, 1995). As is common in many areas of psychology where there is a need and desire to measure unobservable latent constructs, the majority of style assessment methods rely on self-report measures rather than direct objective observation of style-related behavior. The limitations of self-report questionnaire-based measures are well-documented (e.g., Rayner and Riding, 1997) and are particularly pertinent to cognitive style where the prevalent approach to measurement remains single method self-report questionnaires (Cools, 2009). Study designs that utilize multiple methods, including psychometric measures of style and more direct measures of style-based behavior, offer greater potential for validating existing style constructs and construct measures (Cassidy, 2012). However, although the application of a mixed methods approach has the potential to allay some of the limitations associated with self-report questionnaires (Spratt et al., 2004), this approach has been largely overlooked in cognitive styles research (Cools, 2009). One promising area is cognitive neuroscience, with initial findings providing evidence suggesting that cognitive style is directly linked to brain function and behavior. As cognitive style is assumed to reflect underlying cognitive function, evidence linking specific patterns of neural activity to self-report measures of cognitive style would support the validity of such psychometric instruments.

One of the first studies to provide evidence of such a link demonstrated that preferences for visual or verbal cognitive styles were correlated with activity in anatomically and functionally distinct brain regions associated with encoding pictorial (fusiform gyrus) and phonological (supramarginal gyrus; SMG) stimuli, respectively (Kraemer et al., 2009). These findings suggest that individuals who prefer to adopt a visual cognitive style engage in mental imagery of word-based stimuli, and those with a preference for a verbal style show a tendency to verbally encode stimuli even when presented with pictorial information. Further, the results suggest that modality-specific cortical activity underlies processing in visual and verbal cognitive styles.

In a more recent neuroimaging study, Shin and Kim (2015) adopted a modified Stroop task (Stroop, 1935) to investigate whether individual differences in cognitive style influence, through differential responding to distracting information, increases in neural conflict adaptation in brain regions associated with cognitive control. It was evident that the greater the preference for a verbal cognitive style, the greater the conflict adaptation effect. This was especially true for congruent trial types. Furthermore, functional magnetic resonance imaging indicated increased neural conflict adaptation effects in task-relevant brain networks as the preference for a verbal cognitive style increased, suggesting that flexible cognitive control is associated with an individuals' preference for cognitive style (Shin and Kim, 2015).

Whilst these neuroimaging studies are among the first to provide evidence linking preferences in cognitive style to differing patterns of neural activity, they have adopted the visualizer–verbalizer dimension to characterize cognitive and perceptual processing. Research focusing on this characterization of style does not take account of other approaches to cognitive and perceptual processing that reflect the second superordinate orthogonal dimension of cognitive style, *wholist-analyst*, proposed by Riding and Cheema (1991), that includes field-dependent/field-independent (Witkin, 1962) and intuition-analysis (Allinson and Hayes, 1996) approaches. Differences in preferences along these dimensions may impact aspects of cognition including visual attention such that eye-tracking and visual search experiments may offer an additional avenue for styles research.

Eye-tracking provides insight into the spatial and temporal allocation of visual attention and thus holds promise for (1) assessing how cognitive style may relate to which information is prioritized during a visual task, and (2) how cognitive style influences the moment-by-moment process of task completion. Tsianos et al. (2009) demonstrated that visualizers looked more at images whilst verbalizers focussed more on text. Mawad et al. (2015) found that field-independent and field-dependant scores related to which details were prioritized when inspecting food labels. Such studies provide useful behavioral validation for different models of cognitive style in relation to attentional focus. However, we propose that greater insight can be gained by assessing the location *and* temporal order of eye fixations during task completion, as these can reveal how strategy unfolds over time. This is possible because evidence shows that eye fixations pick up information as and when it is used for task completion (Hayhoe and Ballard, 2005). Within cognitive science many studies have applied eye-tracking to understand strategy across a range of tasks, including mental rotation (Just and Carpenter, 1976), visual search (Zelinsky et al., 1997), and comparative visual search (Galpin and Underwood, 2005). However, the focus of this work has been on general patterns in strategy aggregated across participants, rather than individual differences. For example, Galpin and Underwood (2005) demonstrated that observers searched for differences between two pictures by making frequent point-by-point comparisons until detecting a difference, upon which the focus of attention narrowed and fixation durations increased. However, no attempt was made to assess how this strategy varied across participants. We therefore propose that a fruitful line of enquiry would be to assess how such strategies vary in accord with models of cognitive style.

The possibility of combining neuroimaging and/or eye-tracking with visual search paradigms offers a promising avenue for cognitive style research. Visual search tasks can investigate the allocation of attention during task completion (i.e., Galpin and Underwood, 2005; Bendall and Thompson, 2015) and can be combined with neuroimaging techniques (Bendall and Thompson, 2016). Novel non-invasive neuroimaging techniques such as functional near-infrared spectroscopy (fNIRS) have been successfully utilized in a range of cognitive science disciplines (e.g., emotion science; Bendall et al., 2016), and offer a number of advantages including reduced cost, the ability to be employed in a wide range of tasks (e.g., during exercise; Lucas et al., 2012) and enabling data collection from groups otherwise difficult to access such as infants (Franceschini et al., 2007) and clinical populations (Matsubara et al., 2014). These benefits allow for a greater range of tasks to be investigated including those taking place outside of the laboratory. Cognitive styles may be more evident during natural behavior than laboratory tasks, thus portable eye-trackers and fNIRS offer great scope for future research. Further, techniques that do not rely on verbal report could better reveal the development of styles through childhood. Adopting such mixed methods approaches utilizing visual search tasks, eye-tracking, as well as neuroimaging and electrophysiological approaches allows the simultaneous investigation of both overt strategy measures and underlying neural processing, and will aid in revealing the contributions of both strategy and information preference in determining task performance. For instance, it has been argued that the use of event-related potentials can help to reveal the precise information relating to the time course of mental processing that occurs immediately after stimulus (or task) onset (Vanlessen et al., 2016).

We also argue that future cognitive styles research would benefit from not only adopting a mixed method experimental approach, but also from investigating other dimensions of cognitive style beyond the visualizer–verbalizer dimension. For instance, it has been shown that individual differences in brain structure and function are related to preferences in field-dependence/field-independence (Hao et al., 2013) and that field-dependence/field-independence is related to the type of information that is prioritized (Mawad et al., 2015). However, research adopting mixed methods to investigate wholist-analytic dimension of cognitive style is limited.

Whilst some authors argue that cognitive styles are more dynamic than static, so can change or alter (Zang, 2013), others have presented evidence suggesting longer term stability and resistance to modification (Clapp, 1993). Thus, the question of how flexibly a style can be adapted if it is not working, or if a particular mode of task performance is prevented, is not fully resolved. For instance, what if preference for an analytic approach to visual search is discouraged or leads to poorer performance? We argue that an understanding of the underlying neural activity and overt attentional activity will allow the development of paradigms to disrupt preferred cognitive styles and thus assess their flexibility. Initial work in this area has begun to demonstrate that disruption of cognitive style-related brain activity can impact behavior. Targeted transcranial stimulation of the SMG was able to impair performance on a task requiring verbal processing where the scale of this effect was predicted by an individuals' level of verbal cognitive style (Kraemer et al., 2014). One outcome of this line of enquiry may be that, for most people in many scenarios, cognitive styles are habitual modes of processing which can be adapted or over-ridden depending on context. The ultimate aim of validation work in the area of cognitive styles should be to measure behavior in ecologically meaningful activities and settings. This is important as it is plausible that abstract laboratory tasks may encourage participants to focus unnaturally on their own performance leading to artificial behavior that masks habitual cognitive style. Fortunately, “in-the-field” studies are becoming more possible due to advances in technology such as portable fNIRS equipment or unobtrusive and head-mounted eye-tracking equipment. A fully-rounded field of cognitive styles will therefore achieve an understanding of their habitual manifestation, their flexibility and the importance of context in their use. This is only possible through mixed methods research.

A decade has passed since Coffield et al.'s (2004) heavy criticism of the field of cognitive style, based—mainly—on the questionable reliability and validity of self-report psychometric construct measures so often utilized in the field. Despite this, research adopting mixed measures remains scarce. Recently a small number of studies have begun to adopt a neuroscientific approach revealing important findings about behavioral and neural correlates of cognitive style. However, additional mixed methods experimentation is needed to validate the construct of cognitive style, focusing only on those construct measures that are considered valid and reliable, such as the Cognitive Styles Index (Allinson and Hayes, 1996). Additionally, the field stands to benefit from combining various methodologies including neuroimaging and electrophysiology, visual search paradigms, and eye-tracking, whereby information about underlying processing and strategy can be gathered simultaneously. We propose a particularly beneficial avenue for future research moving beyond correlational designs and toward causal experimental designs where disruptions to strategy and processing can be investigated. Whilst mixed-methods afford greater scientific understanding of cognitive styles, it is important to appreciate the practical application of cognitive styles measures in areas in which the need for efficient administration of measurement tools may preclude complex techniques. We are therefore not suggesting the adoption of in the field eye-tracking or neuroimaging by practitioners. Rather, we offer these techniques in response to previous research indicating the need for further work in the area to validate psychometric measures of cognitive style. Adopting the suggested multi-source, multi-method approaches proposed here will provide a valuable contribution in the field of cognitive style measurement.

## Author contributions

RB, opinion concept, main conclusions, article drafting; AG, LM, and SC verification of opinion concept, main conclusions, article drafting.

### Conflict of interest statement

The authors declare that the research was conducted in the absence of any commercial or financial relationships that could be construed as a potential conflict of interest.
